# Prognostic Significance of Echocardiography-Visualized Thrombus in Right Heart in Early Stages of Acute PE: 100 Patients from Regional Pulmonary Embolism Registry

**DOI:** 10.3390/life16071131

**Published:** 2026-07-07

**Authors:** Bojan Mitrovic, M. Radovic, L. Kos, Tamara Kovacevic-Preradovic, B. Dzudovic, S. Salinger, E. Jevtic, V. Miloradovic, I. Mitevska, B. Bozovic, S. Pekovic, J. Matijasevic, A. Biskupovic, Z. Gluvic, S. Kafedzic, A. Neskovic, Slobodan Obradovic

**Affiliations:** 1 Clinic of Internal Medicine, Clinical-Hospital Centre Zemun, 11000 Belgrade, Serbiazorangluvic@yahoo.com (Z.G.);; 2 Faculty of Medicine, University in Belgrade, 11000 Belgrade, Serbia; 3 Clinic of Cardiology, Clinical Center Banja Luka, 78000 Banja Luka, Bosnia and Herzegovinatamara.kovacevic@medicolaser.info (T.K.-P.); 4 Faculty of Medicine, University of Banja Luka, 78000 Banja Luka, Bosnia and Herzegovina; 5 Clinic of Emergency Internal Medicine, Military Medical Academy, 11000 Belgrade, Serbia; 6 Faculty of Medicine, University of Defense, 11000 Belgrade, Serbia; 7 Clinic of Cardiology, Clinical Center Nis, 18000 Nis, Serbia; sonja.salinger@gmail.com; 8 Faculty of Medicine, University of Nis, 18000 Nis, Serbia; 9 Clinic of Cardiology, Clinical Center Kragujevac, 34000 Kragujevac, Serbia; ema.jevtic@gmail.com (E.J.);; 10 Faculty of Medicine, University of Kragujevac, 34000 Kragujevac, Serbia; 11 University Clinic for Cardiology and Cardiovascular Surgery, National Center for Cardiovascular Disease, 1000 Skopje, North Macedonia; 12 University Cardiology Clinic Podgorica, 81000 Podgorica, Montenegro; 13 Institute of Pulmonary Diseases Vojvodina, 21208 Sremska Kamenica, Serbia; 14 Faculty of Medicine, University of Novi Sad, 21000 Novi Sad, Serbia; 15 General Hospital Pancevo, 24000 Pancevo, Serbia; 16 Clinic of Cardiology, Military Medical Academy, 11000 Belgrade, Serbia

**Keywords:** acute pulmonary embolism, thrombus in right heart, mortality, outcome

## Abstract

**Background:** The significance of imaging right-heart thrombus via echocardiography in acute pulmonary embolism (PE) patients, as well as its management, remains uncertain. **Methods:** In this retrospective, observational, multicenter, and multinational registry of consecutive acute PE patients, we compared 100 patients in whom thrombus was visualized in the right heart during echocardiographic evaluation with 2635 patients without visualization of thrombus. The co-primary endpoints were all-cause in-hospital mortality and PE-related mortality. Secondary endpoints included the prevalence of severe PE at admission (intermediate–high and high-risk PE), the frequency of thrombolytic therapy administration, and mortality among patients who received thrombolysis. **Results:** All-cause and PE-related mortality were higher in patients with thrombus in the right heart (31.0% vs. 9.6%, and 25.8% vs. 5.6%, *p* < 0.001, respectively). Patients with right-heart thrombi had a more severe PE presentation than those without (74.0% vs. 45.3%, *p* < 0.001). In patients treated with thrombolysis, all-cause mortality was higher in patients with right-heart thrombi (35.5% vs. 12.7%, *p* < 0.001). In the multivariable Cox regression analysis adjusted for ESC mortality risk, age, presence of syncope, atrial fibrillation, and hypoxemia, patients with thrombus in the right heart had a significantly higher risk of all-cause and PE-related death compared to patients without thrombus (HR 2.60, 95% CI 1.753–3.859, *p* < 0.001, and HR 3.287, 95% CI 2.056–5.253, *p* < 0.001, respectively). **Conclusions:** Visualization of thrombus in transit through the right heart is associated with higher all-cause and PE-related mortality, independent of PE severity and the use of thrombolytic therapy.

## 1. Introduction

Early risk stratification in patients with acute pulmonary embolism (PE) represents the cornerstone of contemporary management, as therapeutic strategy and timely implementation of reperfusion therapy depend directly on risk assessment. The European Society of Cardiology (ESC) 2019 guidelines [1] are based on a four-tiered mortality risk model, encompassing patients at high risk (shock or cardiac arrest), intermediate–high risk (right ventricular dysfunction combined with elevated biomarkers), intermediate-low risk (presence of one of these parameters or an elevated PESI score), and low risk, defined by the absence of all such criteria. This model has become the standard in European practice, as it enables differentiation of patients according to the likelihood of adverse outcomes and guides decisions regarding reperfusion therapy [1].

The most recent American guidelines (AHA/ACC 2026) [2] introduce the concept of Acute Pulmonary Embolism Clinical Categories (A–E), which more precisely define risk and link it to reperfusion recommendations. In this framework, decisions regarding reperfusion therapy are made within a multidisciplinary Pulmonary Embolism Response Team (PERT), beginning with categories C3 through E2, where the need for systemic thrombolysis, catheter-based, or surgical interventions is evaluated [2]. This approach underscores that reperfusion therapy is not uniformly prescribed but rather depends on individualized assessment of clinical status and risk. In practice, therapeutic decisions are made in real time, in intensive care settings, integrating hemodynamic parameters, biomarkers, and echocardiographic findings.

Of particular prognostic importance is the echocardiographic visualization of thrombus within the right heart chambers. This finding is regarded as a marker of acute embolization and is associated with substantially increased in-hospital mortality [3,4,5,6]. Right-heart thrombi may present in several morphologies: free-floating emboli in transit, adherent thrombi attached to the atrial or ventricular wall, and thrombi entrapped within a patent foramen ovale (PFO). Each morphology carries distinct prognostic implications, with free-floating thrombi associated with acute hemodynamic collapse, adherent thrombi linked to persistent obstruction, and PFO-entrapped thrombi carrying the risk of systemic embolization and stroke [3,7,8]. These variants represent a spectrum of critical clinical scenarios, all of which are considered “red flags” requiring urgent intervention in contemporary practice [3,7].

Over the past decade, advanced reperfusion strategies have been developed for the treatment of acute PE, including catheter-based mechanical thrombectomy, local administration of low-dose thrombolytics directly into the thrombus, with or without ultrasound-assisted therapy (EKOS system). The HI-PEITHO study demonstrated that such modalities may improve outcomes in patients with intermediate–high-risk PE, while reducing the risk of major bleeding [9]. These approaches complement standard systemic thrombolysis and surgical embolectomy, offering new opportunities for individualized management in the most critical clinical scenarios [10].

The aim of this study was to investigate, in a large multinational registry of patients with acute PE, the association between the presence of right-heart thrombus and the severity of clinical presentation, in-hospital outcomes, and the frequency and results of thrombolytic therapy in this subgroup. In this way, we sought to define the prognostic significance of echocardiographically visualized right-heart thrombus and its role in guiding therapeutic decisions in contemporary clinical practice [3,4].

## 2. Methodology

This study was designed as a multicenter retrospective analysis of consecutive patients diagnosed with acute pulmonary embolism by computed tomography pulmonary angiography (CTPA) at admission, hospitalized between January 2016 and December 2025. Patients were recruited from a total of eight tertiary and one secondary healthcare institutions in Serbia, Bosnia and Herzegovina, Montenegro and North Macedonia. Patients were admitted to either cardiology units (8 institutions) or pulmonology units. In our registry, the majority of patients were initially treated in intensive or semi-intensive care units in 8 out of 9 participating institutions.

All patients had their diagnosis confirmed by CTPA with thrombus presence required to be documented in at least two subsegmental or one segmental pulmonary artery. According to protocol of the registry, initial echocardiographic evaluation was suggested to all patients, and it was successfully performed in 2735 out of 2797 patients. The remaining 62 patients, for whom echocardiographic data were unavailable, were excluded from the analysis. Echocardiographic examinations were performed according to standardized institutional protocols. All findings were verified by senior cardiologists experienced in echocardiography. In cases of uncertainty, images were re-evaluated by two independent investigators, and discrepancies were resolved by consensus.

Interobserver variability was not formally quantified but is acknowledged as a limitation of the study.

Based on echocardiographic findings, patients were stratified into two groups: those where thrombus (free floating or entrapped) were seen in the right heart, and those without it [3]. For all enrolled patients, demographic data, comorbidities, clinical characteristics, laboratory results, and treatment outcomes were collected followed by the protocol of the registry. Symptoms at admission, vital parameters, biomarkers (cardiac troponins and B-type natriuretic peptides), as well as risk assessment according to the European Society of Cardiology recommended score were determined during the admission process [1]. Every institution has at least one dedicated younger physician who collects data and writes it into the database and a senior investigator who controls the acquisition of all necessary information about the patients. All surviving patients underwent a follow-up visit one month after hospital discharge, while further monitoring was performed according to the local institutional protocols, including regular outpatient controls and assessment of treatment effectiveness.

For the risk stratification we used the 4-stratum Europeans Society of Cardiology (ESC) mortality risk stratification model, which stratified patients into 4 groups: high-risk (patients with acute PE in shock or who had cardiac arrest), intermediate–high-risk (patients with signs of RV dysfunction on echocardiography or CTPA at initial assessment, and elevated blood levels of biomarkers, either cardiac troponins, and/or BNP, and/or NT-proBNP), intermediate-low-risk (patients with RV dysfunction or positive biomarkers, or simplified PESI score 1 or higher), and finally low-risk patients without any of the previously mentioned [1,11,12]. D-dimer levels were measured at admission and are presented as median values with interquartile range (25th–75th percentile). Antithrombin activity was not routinely measured in our registry, and supplementation was not required in any patient.

All participating institutions obtained approval from their respective ethics committees in accordance with current national and international guidelines. The only exception was the Institute of Pulmonology Vojvodina, where approval was granted by the Review Board, serving as the equivalent body for ethical evaluation of research. The study was conducted in accordance with the principles of the Declaration of Helsinki (World Medical Association, 2013) and relevant national regulations.

The patients were informed that their medical data without their identity were recorded into the database of the registry and used for professional and scientific purposes. Form 2025 some institutions required written informed consent for the patient’s involvement in the registry (Military Medical Academy, and University clinical center Zemun).

The co-primary study outcomes were all-cause in-hospital mortality and PE-related mortality. Secondary outcomes comprised the prevalence of severe acute PE (intermediate–high and high-risk according to ESC risk model), the frequency of thrombolytic therapy administration, and mortality among patients who received thrombolysis.

Statistical analysis was performed using standard methods. Continuous variables were presented as mean ± standard deviation and compared using the independent samples t-test, while categorical variables were expressed as absolute and relative frequencies and compared using the χ^2^ test or Fisher’s exact test, where applicable. For multi-category distributions, the likelihood ratio test was applied. The association of right-heart thrombus with PE severity, mortality outcomes, and thrombolysis use was assessed by univariate and multivariable regression analyses, including the Cox proportional hazards model for all-cause and PE-related hospital mortality, and logistic binary regression for the severity of PE, and use of thrombolysis, as an dependent variables. PE-related mortality was defined as death most probably caused by acute PE shock, according to the attending physician’s judgment at each participating center. This pragmatic definition is consistent with standardized recommendations proposed by the ISTH SSC [13]. Thrombolysis and other reperfusion techniques were included as covariates in the multivariable regression models to account for potential confounding by treatment allocation. Time-to-event data were available, and Cox proportional hazards models were applied to account for variable lengths of hospital stay. The proportional hazards assumption was tested using Schoenfeld residuals and graphical inspection, and no significant violations were observed. Results were reported as odds ratios (OR), or hazard ratios (HR) with 95% confidence intervals, and statistical significance was defined as *p* < 0.05.

## 3. Results

A total of 2735 patients with acute pulmonary embolism were included in the study. Right-heart thrombus was documented in 100 patients (3.7%), while the remaining 2635 patients (96.3%) had no evidence of thrombus.

Demographic data and comorbidities are presented in Table 1. No significant differences were observed between the groups, except that patients with right-heart thrombus were significantly younger.

The main symptoms, clinical manifestations, and laboratory markers are presented in Table 2. Patients with right-heart thrombus had a significantly higher frequency of syncope (35.0% vs. 13.8%; *p* < 0.001), resuscitation at admission (20.0% vs. 5.3%; *p* < 0.001), hypotension with systolic BP < 100 mmHg (30% vs. 10%; *p* < 0.001), tachycardia ≥ 110/min (52% vs. 30%; *p* < 0.001), abnormal oxygen saturation (51.8% vs. 27.1%; *p* < 0.001), as well as elevated biomarkers (BNP > 100: 82.0% vs. 60.4%; *p* = 0.002; troponin: 77.2% vs. 56.4%; *p* = 0.001). Patients with thrombus in the right heart had significantly higher D-dimer levels (median 8787 ng/mL; IQR 4851–19,795) compared to patients without thrombus (median 4200 ng/mL; IQR 2200–8662, *p* < 0.001). These values are presented in Table 2. Time from the symptoms onset to admission to hospital was shorter in patients with right-heart thrombi than in patients without is.

Regarding the co-primary outcomes, all-cause in-hospital mortality was significantly higher in patients with right-heart thrombus compared with those without (31.0% vs. 9.6%; *p* < 0.001), and PE-related mortality was also markedly higher (25.8% vs. 5.2%; *p* < 0.001). As secondary outcomes, patients with right-heart thrombus had a significantly higher prevalence of intermediate–high and high-risk PE (74.0% vs. 45.2%; *p* < 0.001). A detailed overview of these differences is presented in Table 3.

Advanced reperfusion therapy was applied in 68.0% of patients with thrombus in right heart, and in 18.6% of patients without it (Table 3). Among six patients treated with mechanical thrombectomy in the group with thrombus in the right heart, two of them were treated with surgical thrombectomy because of a thrombus entrapped in the opened foramen ovale, and in three of them surgical intervention was done because of a very large thrombus entrapped in the trabeculae of the right heart. Only one patient in this group was treated with catheter mechanical thrombectomy after the thrombus passed to the pulmonary arteries and the patients had absolute contraindication for systemic thrombolysis.

As secondary outcomes, thrombolytic therapy was more frequently administered in patients with right-heart thrombus (62.0% vs. 18.3%; *p* < 0.001). However, despite the more frequent use of reperfusion therapy, mortality after thrombolysis was significantly higher in the thrombus group (35.5% vs. 12.7%; *p* < 0.001). PE-related mortality among patients who received thrombolysis was also markedly higher in the thrombus group (32.2% vs. 8.5%; *p* < 0.001). These findings indicate that the presence of right-heart thrombus represents a marker of more severe clinical presentation and poorer prognosis. In addition to reperfusion therapy, more than 70% of patients in both groups received low-molecular-weight heparin (LMWH) during hospitalization, while the remaining patients were treated with unfractionated heparin in the first few days. Subsequently, almost all patients were transitioned to direct oral anticoagulants (DOACs) for long-term therapy.

In univariable analysis, the presence of right-heart thrombus significantly increased the risk of severe PE (OR 3.747; 95% CI 2.353–5.968; *p* < 0.001), all-cause in-hospital mortality (HR 3.728; 95% CI 2.567–5.414; *p* < 0.001), PE-specific mortality (HR 5.592; 95% CI 3.618–8.643; *p* < 0.001), and the likelihood of thrombolysis use (OR 4.121; 95% CI 2.516–6.751; *p* < 0.001). The risk of mortality among patients who received thrombolysis was also significantly higher in the thrombus group (HR 3.146; 95% CI 1.868–5.299; *p* < 0.001).

In univariable analysis, the presence of right-heart thrombus significantly increased the risk of the co-primary outcomes: all-cause in-hospital mortality (HR 3.728; 95% CI 2.567–5.414; *p* < 0.001) and PE-specific mortality (HR 5.592; 95% CI 3.618–8.643; *p* < 0.001). As secondary outcomes, right-heart thrombus was associated with a higher prevalence of severe PE (OR 3.747; 95% CI 2.353–5.968; *p* < 0.001), greater likelihood of thrombolysis use (OR 4.121; 95% CI 2.516–6.751; *p* < 0.001), and increased mortality among patients who received thrombolysis (HR 3.146; 95% CI 1.868–5.299; *p* < 0.001). In multivariable analysis, adjusted for age and PE severity, right-heart thrombus remained an independent predictor of adverse outcomes. After adjustment for reperfusion therapy, right-heart thrombus remained an independent predictor of all-cause and PE-related mortality. The risk for all-cause mortality was HR 2.601 (95% CI 1.753–3.859; *p* < 0.001), and for PE-related mortality HR 3.287 (95% CI 2.056–5.253; *p* < 0.001). As secondary outcomes, RHT was also associated with severe PE (OR 3.453; 95% CI 2.138–5.579; *p* < 0.001), higher likelihood of thrombolysis (OR 3.551; 95% CI 2.078–6.067; *p* < 0.001), and increased mortality among patients treated with thrombolysis (HR 2.556; 95% CI 1.645–3.927; *p* < 0.001). The results of the univariable and multivariable regression analyses are presented in Table 4.

## 4. Discussion

The results of our multicenter study clearly demonstrate that the presence of right-heart thrombus (RHT) in patients with acute pulmonary embolism (PE) is associated with significantly higher all-cause and PE-related in-hospital mortality as co-primary outcomes, and with more severe clinical presentation, greater use of thrombolysis, and higher mortality despite reperfusion therapy as secondary outcomes, independent of the risk estimated by the ESC mortality score [1,2,3]. In our cohort, patients with RHT had a significantly higher prevalence of syncope, need for resuscitation at admission, hypoxemia, and elevated biomarkers, all of which were accompanied by a markedly greater frequency of intermediate–high and high-risk PE [4,5]. We also observed that patients with RHT more frequently presented with hypotension (systolic blood pressure < 100 mmHg) and tachycardia ≥ 110/min, further confirming their clinical instability and explaining the higher mortality in this subgroup [6]. In addition, patients with right-heart thrombus had significantly higher D-dimer levels compared with those without thrombus. Elevated D-dimer in this subgroup may reflect an ongoing prothrombotic state, although ranges overlap, and therefore should be interpreted with caution. We acknowledge that antithrombin activity was not assessed in our registry. However, none of the patients required antithrombin supplementation during hospitalization, which we consider an important limitation of the study. These findings are consistent with previous studies indicating that RHT (“embolus in transit”) denotes an acute, unstable event with a high risk of sudden deterioration and fatal outcome [1,8,9].

The largest published series of patients with RHT originates from the RIETE registry, where among 12,441 patients with acute PE, 325 were identified with RHT (2.6%) [10,13]. Mortality in that cohort was lower than in our series, which may be explained by the fact that our patients had a more severe clinical presentation and were predominantly treated in intensive care units. Nevertheless, even in the RIETE registry, the presence of RHT proved to be an independent predictor of adverse outcome [11]. Our multicenter analysis, with 100 patients presenting with RHT, represents one of the largest single series after RIETE and Watson’s multicenter U.S. cohort (345 patients) [3]. RIETE remains the largest database, while Watson et al. provide the most extensive series focused exclusively on RHT. Our analysis positions itself immediately after these studies, but with a particularly severe population treated in intensive and semi-intensive care units, offering unique insight into the most critical subgroup [4,12,14]. The unfavorable outcomes in these patients can be explained by the acute nature of the event, where the organism has no time to develop compensatory mechanisms. Unlike chronic conditions, in which the right ventricle gradually adapts to increased pulmonary pressure, acute massive embolism leads to a sudden rise in afterload and rapid decompensation. This situation is analogous to acute valvular insufficiencies (mitral and aortic regurgitation), which carry a significantly worse prognosis compared with chronic forms precisely due to the absence of adaptive mechanisms [15,16]. Our results confirm that the presence of RHT marks a “critical point” in the pathophysiological course of PE, with a substantially increased risk of fatal outcome [17,18].

According to current European (ESC 2019) and American (AHA, ASH 2026) guidelines, patients with RHT are classified as high-risk and require a more aggressive therapeutic approach [1,2]. The AHA/ACC 2026 guidelines emphasize that treatment decisions in these patients should be made by a multidisciplinary Pulmonary Embolism Response Team (PERT), taking into account right ventricular function, thrombus size and position, and bleeding risk [19]. RHT in transit is specifically designated as a high-risk situation, where urgent reperfusion therapy is strongly recommended. Standard anticoagulation (heparin, LMWH, DOACs) is mandatory but likely insufficient in this subgroup. Systemic thrombolysis is recommended as first-line therapy in hemodynamically unstable patients, and in the presence of RHT it should be considered even in the absence of overt instability, given the high risk of sudden deterioration [1,20,21]. An important exception is thrombus entrapped in a patent foramen ovale (PFO), where systemic thrombolysis may cause uncontrolled fragmentation and systemic embolization, including ischemic stroke [22]. Catheter-based interventions (aspiration thrombectomy, local thrombolysis) represent alternatives in patients with contraindications to systemic thrombolysis or in centers with appropriate expertise [23]. Surgical embolectomy is reserved for massive thrombi or failure of other methods, but published data suggest it may be the treatment of choice in cases of large RHT [3,24].

In our REPER registry, we identified 100 patients with RHT at admission. We demonstrated that visualization of RHT is an independent risk factor for both all-cause and PE-specific mortality, regardless of ESC risk, age, syncope, hypoxemia, or new-onset atrial fibrillation. The presence of RHT was associated with more severe clinical presentation and more frequent use of reperfusion therapy. Our data show that patients with RHT were more often treated with thrombolysis, yet mortality despite reperfusion remained significantly higher compared with patients without RHT [3,11,17]. Nevertheless, residual confounding by indication cannot be fully excluded, as reperfusion therapy was more frequently administered to critically ill patients. Accordingly, the finding that mortality remained higher in RHT patients despite thrombolysis should be interpreted with caution, since thrombolytic therapy was preferentially given to those with significantly worse baseline clinical status. Although the follow-up period was short and homogeneous, Cox regression was appropriate given the availability of time-to-event data and variable lengths of hospital stay. The proportional hazards assumption was formally tested using Schoenfeld residuals and graphical inspection, and no significant violations were observed. This methodological approach allowed us to provide hazard ratios that reflect instantaneous risk, complementing logistic regression analyses of secondary outcomes. We also acknowledge that PE-related mortality was adjudicated by attending physicians, which may introduce inter-center variability and misclassification. This limitation has been recognized in previous work, and standardized definitions have been proposed by the ISTH SSC to improve reproducibility and comparability across studies [13]. Similar findings were reported by Watson et al. in a multicenter U.S. cohort, where mortality in RHT patients was significantly higher than in those without thrombus, confirming RHT as an independent predictor of poor outcome [3]. In supplementary analyses, the presence of RHT provided incremental prognostic information beyond ESC risk stratification, particularly in high-risk and intermediate–high-risk PE subgroups.

Shorter time from symptom onset to hospitalization in our cohort was significantly associated with the presence of RHT, consistent with literature indicating that rapid symptom progression denotes more severe clinical presentation and worse prognosis [3,16,19]. The presence of thrombus entrapped in a PFO may lead to systemic embolization and stroke, particularly after systemic thrombolysis [22].

Our results confirm that RHT is not only a marker of more severe clinical status but also a predictor of poorer response to reperfusion therapy. This underscores the need for early recognition of these patients and decision-making within a multidisciplinary PERT to individualize therapeutic strategies and optimize outcomes [2,19]. The presence of RHT should be regarded as a “red flag” in clinical practice, requiring urgent intervention and consideration of reperfusion modalities. Our findings are consistent with the work of Dzudović et al. who demonstrated that mortality in patients with RHT in transit reaches 20–40%, regardless of treatment modality [8]. The authors emphasized that anticoagulation alone carries the worst outcomes, while thrombolysis, catheter-based interventions, and surgical embolectomy may improve prognosis, although mortality remains substantial [9,20,24].

### Limitations of the Study

This study has several important limitations. The first of them is that echocardiography examination was performed in many patients during outside working hours by the cardiologists on duty, and many of them are not experts in echocardiography and are trained only for focus examination. In this way, some examinations might have missed smaller thrombi in the right heart, or in contrary some structures might have been misinterpreted as thrombi. Since in the majority of patients echocardiography imaging or right-heart thrombi was re-evaluated by the wider team of doctors in the next working day, we assumed that the number of false negative and false positive results were minimal. The second limitation is the difference in the institutions involved in the facilities and expertise for the treatment of acute PE patients. Namely, only 3 out of 10 hospitals involved had Pulmonary Embolism Response Teams, for at least part of the investigation, and those institutions also could perform catheter-directed therapy; only one institution could organize 24/7 surgical thrombectomy. However, our investigation is registry-based, and this kind of organization represents a mix of hospitals with different capabilities which are all involved in the emergency treatment of acute PE. The third limitation is the use of PE-related death as a co-primary endpoint, which was left at the discretion of every attending physician, who decided how to classify the cause of death if a patient died in refractory shock or suddenly, which was most likely caused by acute PE during the index hospitalization.

## 5. Conclusions

In summary, the presence of right-heart thrombus in patients with acute pulmonary embolism was associated with significantly higher all-cause and PE-related in-hospital mortality as co-primary outcomes. In addition, right-heart thrombus was identified patients with more severe clinical presentation, greater need for thrombolysis, and higher mortality despite reperfusion therapy as secondary outcomes. These findings highlight right-heart thrombus as a strong prognostic marker that should be recognized early in clinical practice, independent of conventional risk stratification models, and may guide therapeutic decision-making in this high-risk population.

REPER is an academic registry and authors have nothing to declare considering participation in the registry and the writing of this manuscript.

## Figures and Tables

**Table 1:** Demographic characteristics and comorbidities according to the presence of right-heart thrombus.

Variable	Thrombus in RH Yes *n* (%)	Thrombus in RH No *n* (%)	*p*-Value
**Age, mean ± SD**	63 ± 16	68 ± 16	**0.034**
**Sex (M/F)**	46 (46.0%)/54 (54.0%)	1249 (47.4%)/1386 (52.6%)	0.783
**Age > 74 years**	23 (23.0%)	724 (27.5%)	0.324
**COPD/chronic lung disease**	10 (10.0%)	292 (11.0%)	0.759
**Major surgery**	19 (19.0%)	419 (15.9%)	0.411
**Active malignancy**	16 (16.0%)	411 (16.2%)	0.806
**Hypertension**	61 (61.0%)	1620 (61.6%)	0.997
**Coronary artery disease**	17 (17.0%)	347 (13.2%)	0.277
**Diabetes mellitus**	20 (20.0%)	549 (20.9%)	0.876
**Previous stroke**	6 (6.0%)	200 (7.6%)	0.569
**Previous bleeding**	5 (5.0%)	145 (5.3%)	0.416

RH—right heart; SD—standard deviation.

**Table 2:** Clinical presentation, risk, and laboratory markers according to the presence of right-heart thrombus.

Variable	Thrombus in RH—Yes (n = 100)	Thrombus in RH—No (n = 2635)	*p*-Value
**Dyspnea**	89 (89.0%)	2161 (82.0%)	0.082
**Chest pain**	31 (31.0%)	1025 (38.9%)	0.098
**Hemoptysis**	6 (6.0%)	170 (6.5%)	0.531
**Syncope**	35 (35.0%)	363 (13.8%)	**<0.001**
**Resuscitation at admission**	20 (20.0%)	140 (5.3%)	**<0.001**
**Symptomatic DVT**	30 (30.0%)	881 (33.4%)	0.660
**Time to admission ≤ 24 h**	46 (46.0%)	871 (33.1%)	0.015
**Time of symptom onset to admission in hours**	48 (12–72)	72 (24–120)	0.001
**PESI > 0**	87 (87.0%)	2086 (79.2%)	0.165
**Risk categories (low/int-low/int-high/high)**	10 (10.0%)/16 (16.0%) /38 (38.0%)/36 (36.0%)	766 (29.1%)/677 (25.7%)/810 (30.7%)/381 (14.5%)	**<0.001**
**AF (sinus/paroxysmal/permanent)**	81 (82.7%)/12 (12.2%)/5 (5.1%)	2136 (85.3%)/173 (6.9%)/194 (7.8%)	**0.075**
**New-onset AF**	13 (13.1%)	181 (7.2%)	**0.025**
**Systolic BP < 100 mmHg**	30 (30.0%)	275 (10.4%)	**<0.001**
**Heart rate ≥ 110/min**	52 (52.0%)	795 (30.3%)	**<0.001**
**Abnormal O_2_ saturation**	40 (51.9)	524 (27.1%)	**<0.001**
**BNP > 100**	50 (82.0%)	810 (60.4%)	**0.002**
**Elevated troponin**	61 (77.2%)	1054 (56.4%)	**0.001**
**Positive biomarker (BNP and/or Tn)**	75 (84.3%)	1391 (65.2%)	**0.001**
**D-dimer (median, IQR)**	8787 (4851–19.795)	4200 (2200–8662)	**<0.001**

RH—right heart; DVT—deep vein thrombosis; PESI—pulmonary embolism severity index; BNP—B-type natriuretic peptide; Tn—troponin; AF—atrial fibrillation.

**Table 3:** The presence of thrombus in the right heart and study outcomes: co-primary outcomes (all-cause and PE-related mortality) and secondary outcomes (severity of PE, use of thrombolysis, and mortality among patients who received thrombolysis).

Outcome	Thrombus in RH	No Thrombus in RH	*p*-Value
**Intermediate–high and high-risk PE** ** N/N_all_,** ** %**	74/100 **(74.0%)**	1191/2634 **(45.3%)**	**<0.001**
**All-cause mortality** ** N/N_all_, ** ** %**	31/100 **(31.0%)**	252/2635 **(9.6%)**	**<0.001**
**PE-related mortality** ** N/N ^1^,** ** %**	24/93 **(25.8%)**	131/2525 **(5.2%)**	**<0.001**
**Thrombolysis** ** N/N ^2^, ** ** %**	62/100 **(62.0%)**	480/2619 **(18.3%)**	**<0.001**
**Mortality on thrombolysis** ** N/N ^3^, ** ** %**	22/62 **(35.5%)**	61/480 **(12.7%)**	**<0.001**
**PE-related mortality on thrombolysis N/N ^1^,** **%**	19/59 **(32.2%)**	39/460 **(8.5%)**	**<0.001**

^1^ Denominators for PE-related mortality refer only to patients whose cause of death was assessed as PE; patients who died from other causes were excluded. ^2^ Patients who presented as intermediate–high and high-risk PE. ^3^ Patients who were treated with thrombolytic therapy.

**Table 4:** Thrombus in the right heart and risk for study outcomes: co-primary outcomes (all-cause and PE-related hospital death) and secondary outcomes (PE severity, thrombolysis use, and mortality among patients treated with thrombolysis).

Risk for Endpoints	Univariant Analysis	Multivariable Analysis
**Risk for severe PE (intermediate–high, and high-risk PE)** **(OR, 95% CI, *p*)**	3.747 (2.353–5.968, *p* < 0.001)	3.453 (2.138–5.579) ^1^
**Risk for all-cause hospital death** **(HR, 95% CI, *p*)**	3.728 (2.567–5.414, *p* < 0.001)	2.601- (1.753–3.859) ^2^
**Risk for PE-related hospital death** **(HR, 95% CI, *p*)**	5.592 (3.618–8.643, *p* < 0.001)	3.287 (2.056–5.253) ^2^
**Risk to receive thrombolytic therapy ^3^** **(OR, 95% CI, *p*)**	4.121 (2.516–6.751, *p* < 0.001)	3.551 (2.078–6.067) ^1^
**Risk of all-cause hospital death among patients who were treated with thrombolysis**	3.146 (1.868–5.299, *p* < 0.001)	2.556 (1.645–3.927) ^2^
**Risk for PE-related hospital death on thrombolysis (HR, 95% CI, *p*)**	5.592 (3.618–8.643, *p* < 0.001)	2.839 (1.659–4.861) ^2^

^1^ Adjusted to age. ^2^ Adjusted to age, PE severity in four stratums, syncope, arterial Oxygen saturation < 90% on room air, and the presence of new-onset AF. ^3^ Risk is calculated only in patients with intermediate–high and high-risk PE.

## Data Availability

All the data from the registry are available for everyone who kindly request them.
